# β-Amyloid–Dependent and –Independent Genetic Pathways Regulating CSF Tau Biomarkers in Alzheimer Disease

**DOI:** 10.1212/WNL.0000000000200605

**Published:** 2022-08-02

**Authors:** Atul Kumar, Shorena Janelidze, Erik Stomrud, Sebastian Palmqvist, Oskar Hansson, Niklas Mattsson-Carlgren, for the Alzheimer's Disease Neuroimaging Initiative

**Affiliations:** From the Clinical Memory Research Unit (A.K., S.J., E.S., S.P., O.H., N.M.-C.), Department of Clinical Sciences, Lund University, Malmö; Memory Clinic (E.S., S.P., O.H.), Skåne University Hospital, Malmö; Department of Neurology (N.M.-C.), Skåne University Hospital, Lund; and Wallenberg Centre for Molecular Medicine (N.M.-C.), Lund University, Sweden.

## Abstract

**Background and Objectives:**

Abnormal metabolism of β-amyloid (Aβ) and soluble phosphorylated tau (P-tau), as well as neurodegeneration, are key components of Alzheimer disease (AD), but it is unclear how these different processes are related to genetic risk factors for AD.

**Methods:**

In the Swedish BioFINDER study, we tested associations between a priori defined polygenic risk scores (PRSs) for AD (excluding single-nucleotide polymorphism [SNP] within the *APOE* region in the main analysis) and biomarkers in CSF (total tau [T-tau] and P-tau181; Aβ1-38, Aβ1-40, Aβ1-42, and Aβ1-42/1-40; and neurofilament light [NfL]) in cognitively unimpaired (CU) individuals (n = 751), and in patients with mild cognitive impairment (MCI) (n = 212) and AD dementia (n = 150). Results were validated in the Alzheimer's Disease Neuroimaging Initiative data set with 777 individuals (AD = 119, MCI = 442, and CU = 216).

**Results:**

PRSs with SNPs significant at *p* < 5e-03 (∼1,742 variants) were associated with higher CSF P-tau181 (β = 0.13, *p* = 5.6e-05) and T-tau (β = 0.12, *p* = 4.3e-04). The associations between PRS and tau measures were partly attenuated but remained significant after adjusting for Aβ status. Aβ pathology mediated 37% of the effect of this PRS on tau levels. Aβ-dependent and Aβ-independent subsets of the PRS were identified and characterized. There were also associations between PRSs and CSF Aβ biomarkers with nominal significance, but not when corrected for multiple comparisons. There were no associations between PRSs and CSF NfL.

**Discussion:**

Genetic pathways implicated in causing AD are related to altered levels of soluble tau through both Aβ-dependent and Aβ-independent mechanisms, which may have relevance for anti-tau drug development.

Alzheimer disease (AD), the most common neurodegenerative disease, is characterized by the accumulation of β-amyloid (Aβ) plaques, tau tangles,^1^ neurodegeneration, and cognitive loss.^2-4^ Different pathophysiologic processes can be monitored in AD using biomarkers in CSF and plasma.^5,6^

Several hereditary, behavioral, and environmental influences affect the risk for AD. A few cases have Mendelian inheritance trends, which often result in the early onset of symptoms through altered metabolism of Aβ,^7^ but for most patients, the genetic predisposition is more complex.^8^ The most common genetic risk factor is variants of the *APOE* gene,^9^ which is believed to mainly increase the risk for AD through modulating the accumulation of Aβ.^10^ In addition, genome-wide association studies (GWASs) have identified additional single-nucleotide polymorphisms (SNPs) with risk effects for AD dementia. Still, it remains unclear which of the different key pathophysiologic processes in AD are mainly affected by the many SNPs with low or medium effect sizes for AD risk.

Multiple genetic risk variants, with a minor individual contribution to disease risk, can be combined in polygenic risk scores (PRSs). This has been used to forecast the probability of neurologic diseases with complex traits such as schizophrenia and bipolar disorder.^11^ Such scores combine genome-wide knowledge to compensate for the phenotypic heterogeneity found in specific traits by suggesting that several variants of small impact sizes have a cumulative, nonmultiplicative effect.

This study aimed to test associations between genetic risk factors for AD (beyond *APOE*) and biomarkers reflecting abnormal metabolism of Aβ, soluble phosphorylated tau (P-tau), and neurodegeneration to understand different aspects of AD pathophysiology using CSF biomarkers as proxies for relevant brain changes. We used a priori defined PRSs based on the results of a recent major AD meta-analysis (consisting of 21,982 late-onset AD cases and 41,944 cognitively normal controls)^12^ and tested them in a cohort of cognitively unimpaired (CU) individuals as well as patients with mild cognitive impairment (MCI) and AD dementia in the BioFINDER study. In addition, we validated our findings in the Alzheimer's Disease Neuroimaging Initiative (ADNI) data set.

## Methods

### Standard Protocol Approvals, Registrations, and Patient Consents

The Regional Ethics Committee in Lund, Sweden, approved the BioFINDER study. All participants gave written informed consent. The local ethical committees of all involved sites gave ethical approval in ADNI.

### Study Participants

The study included 751 CU older adults, 212 patients with MCI, and 149 patients with AD dementia from the Swedish BioFINDER sample (Clinical Trial No. NCT01208675),^13^ for whom age, education, sex, and biomarker data were available. Details about recruiting have previously been provided,^14,15^ and the supplement contains additional information. Following the research guidelines,^16^ the CU group consisted both normal controls (N = 569) and patients with subjective cognitive decline (N = 182).

### Validation Sample

We validated parts of the findings in participants (CU, MCI, and AD) from the ADNI (using the phases ADNI-1, ADNI-GO, and ADNI-2).^17^ CSF total tau (T-tau), P-tau181, and Aβ1-42 biomarker data were available for 986 ADNI participants (AD = 186, MCI = 510, and CU = 290) of European ancestry (Supplement contains additional information). To prevent overfitting due to the nonindependence of the GWAS discovery sample and the target sample, 209 ADNI participants who were part of the discovery sample^12^ (used to generate the PRS) were omitted before the PRS estimation, resulting in a final sample of 777 individuals (AD = 119, MCI = 442, and CU = 216).

### Genotyping and Preparation of Genetic Data

For genotyping, the Illumina platform GSA-MDA v2 was used. Quality control (QC) was performed at the subject and SNP levels according to established protocols.^18^ Person-based QC included consistency between chip-inferred and self-reported sex, call rates (1% cutoff), and intense heterozygosity. In addition, high-quality variants (autosomal, biallelic variants with Hardy-Weinberg equilibrium *p* > 5e-08, minor allele frequency [MAF] ≥5%, and a call rate of >99%) were used. Similar QC was applied for the ADNI participants. The supplement provides more information on the imputation and QC for both genetic data.

### Fluid Biomarkers

CSF handling followed a structured preanalytical protocol.^19^ CSF Aβ peptides (including Aβ1-42, Aβ1-40, and Aβ1-38), T‐tau, and P‐tau181 were analyzed using Euroimmun immunoassays (EUROIMMUN AG, Lübeck, Germany), as previously described.^20^ A pathologic Aβ status was defined as CSF Aβ1-42/Aβ1-40 >0.091.^21^ CSF neurofilament light (NfL) concentration was determined using a sensitive sandwich ELISA method (NF-light ELISA kit; UmanDiagnostics AB, Ume, Sweden), as previously described.^22,23^

CSF samples' collection and handling in ADNI are described elsewhere.^24^ In brief, CSF T-tau, P-tau181, and Aβ1-42 in ADNI were measured using the Elecsys immunoassay at the Biomarker Research Laboratory, University of Pennsylvania, Philadelphia, according to the preliminary kit manufacturer's instructions and as described in previous studies.^25^

### Polygenic Score Calculation

Using the weighted effect for each SNP, the PRS was determined using PLINK2.^26^ SNPs were pruned using PLINK's clump function with an *r*^2^ < 0.1 over 1,000 kb pairs before PRS estimation. *APOE* is the most well-known risk factor for AD, with high levels of linkage disequilibrium in the area surrounding the locus. Therefore, when generating the PRS for AD, SNPs falling within the *APOE* region (chr19:44400000-46500000; GRCh37/hg19 assembly) were omitted from the data set. In addition, to test how *APOE* status might affect the significance of the identified PRSs, we also generated PRS models that included the *APOE* region variants. To define PRS for AD, we used publicly available summary statistics from published GWAS studies (not overlapping with the BioFINDER data set).^12^ To determine acceptable *p*-value thresholds, we iterated over a range of values (*p* < 0.05 to *p* < 5e-08) to generate PRS1-7 models (e.g., PRS1 includes all variants significant at *p* < 0.05; details given in eMethods, links.lww.com/WNL/C22).

### Identification of the Tau-Specific PRS Variants That Are Independent of Aβ vs Dependent on Aβ

We used a heuristic approach to generate PRS components associated with tau biomarkers, independent of Aβ and dependent on Aβ. For this, we first created “n” different PRSs (n = number of variants in full PRS) by removing 1 particular variant [“i”], leaving “n−1” variants. We next tested whether the effect of these pruned PRSs on tau biomarkers was mediated by Aβ status. The pruned PRSs were arranged in the ascending order of *p* value of association between the independent variable (PRS) and Aβ (the top PRS was the most strongly associated with Aβ in the absence of ith variant). Using this ranked list of variants, we recreated “n” different PRSs, with an ascending number of variants (the first PRS only included the top variant, the second PRS had the top 2 variants, the third PRS the top 3 variants, and so on), and again used mediation analysis to measure how much of the effect of each of the new increasingly complex PRSs on tau biomarkers that were mediated by Aβ status. This approach identified novel PRSs having strong associations with tau biomarkers that were Aβ-independent. We followed a similar approach to identify novel PRSs that had effects on tau biomarkers dependently on Aβ by repeating the procedure but arranging the variants in descending order of *p* value of association between PRS and Aβ.

### Statistical Analyses

We used linear regression models to investigate the relationship of PRSs with biomarker levels. The biomarkers were rank-based inverse normal transformed and used as dependent variables in linear regression models, adjusted for the covariates' age, sex, education, *APOE* ε4 and ε2 counts (0, 1, 2) [not for PRS including the APOE region variants], mini-mental state examination, and the top 10 principal components from the principal component analysis on the entire set of genotype data. In addition, logistic regression models were used for the PRS on dichotomized biomarkers (including the same covariates).

We used bootstrapping techniques to assess the indirect effect in mediation analysis (n = 1,000 bootstrap samples). Each set of association analyses was corrected for a family-wise error rate using Bonferroni correction. Associations below a Bonferroni corrected *p* value of 0.05 were considered significant. All the statistical analysis was conducted in R programming (version 4.0.2).

### Data Availability

Anonymized BioFINDER data will be shared by request from a qualified academic investigator for the sole purpose of replicating procedures and results presented in the article if data transfer agrees with EU legislation on the general data protection regulation and decisions by the Swedish Ethical Review Authority and Region Skåne, which should be regulated in a material transfer agreement.

Genome-wide summary statistics used to generate Alzheimer PRS can be downloaded from the National Institute on Aging Genetics of Alzheimer's Disease Data Storage Site—an NIA/NIH-sanctioned qualified-access data repository, under accession NG00075. For ADNI data: Data is stored (publicly available) at the LONI database.^27^

## Results

The demographic information on the study population is summarized in Table 1. In addition, the available sample size (based on the diagnostic group) for different biomarkers is given in eTable 1 (links.lww.com/WNL/C21).

**Table 1:**
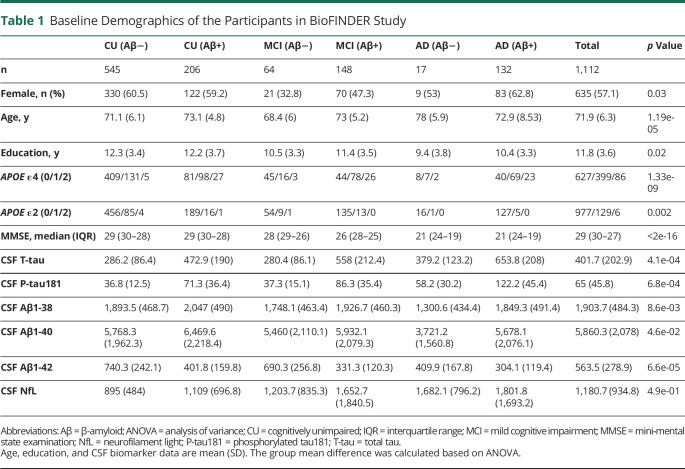
Baseline Demographics of the Participants in BioFINDER Study

### Association Between PRS and Tau Measures

**Figure 1:**
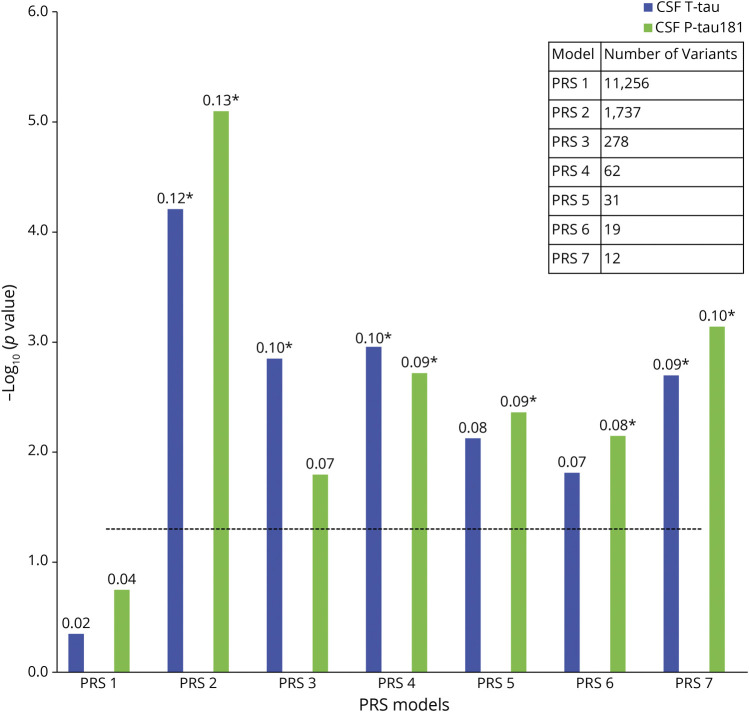
Associations Between PRSs and Tau Measures The x-axis represents the 7 different PRS models at different *p* value thresholds based on the GWAS summary statistics (PRS1 ≤ 0.05, PRS2 ≤ 5e-3, PRS3 ≤ 5e-4, PRS4 ≤ 5e-5, PRS5 ≤ 5e-6, PRS6 ≤ 5e-7, and PRS7 ≤ 5e-8). The models were adjusted for age, sex, education, baseline MMSE, *APOE* ε2 and ε4 count, and the top 10 principal components from the principal component analysis on the entire set of genotype data. The y-axis shows the negative log of the *p* value for the significance of associations between PRS models with different tau measures. The values on the top of each bar show the association's effect size (β-coefficient). The horizontal dotted line shows the *p* value threshold of 0.05. *These PRSs were significant after Bonferroni correction at *p* value <0.05. GWAS = genome-wide association study; MMSE = mini‐mental state examination; PRS = polygenic risk score.

We first tested associations between PRS (excluding *APOE* region variants [non-*APOE*-PRS]) and CSF T-tau and P-tau181 in the BioFINDER study population. PRS2 (including 1,742 SNPs significant at *p* < 5e-03 in the original GWAS for AD dementia vs controls^12^) showed the strongest association with both CSF T-tau (*p* = 4.3e-04) and CSF P-tau181 (*p* = 5.6e-05). It was followed by PRS4 (including 63 SNPs significant at *p* < 5e-05) showing significant associations with CSF T-tau (*p* = 7.8e-03) and CSF P-tau181 (*p* = 1.3e-02). In addition, PRS3 (including 279 SNPs significant at *p* < 5e-04) and PRS7 (including 12 SNPs significant at *p* < 5e-08) were significantly associated with T-tau (*p* = 9.9e-03 and 1.4e-02, respectively), whereas PRS5 (including 31 SNPs significant at *p* < 5e-06), PRS6 (including 19 SNPs significant at *p* < 5e-07), and PRS7 were significantly associated with CSF P-tau181 (*p* = 3.1e-02, 5e-02, and 5.1e-03, respectively) (Figure 1; eTables 2 and 3, links.lww.com/WNL/C21).

We also tested the association between CSF T-tau and P-tau181 and PRS, including the APOE region variants (*APOE*-PRS). All the PRSs showed significant association with T-tau and P-tau181 with a *p* value <1.2e-05 (eTables 2 and 3, links.lww.com/WNL/C21).

### Association Between PRS and Aβ Measures

**Figure 2:**
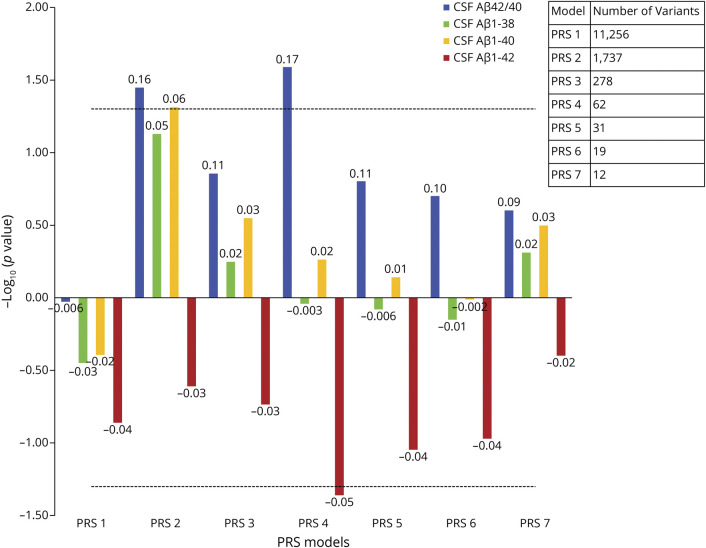
Associations Between PRSs and Aβ Measures The x-axis represents the 7 different PRS models at different *p* value thresholds based on the GWAS summary statistics (PRS1 ≤ 0.05, PRS2 ≤ 5e-3, PRS3 ≤ 5e-4, PRS4 ≤ 5e-5, PRS5 ≤ 5e-6, PRS6 ≤ 5e-7, and PRS7 ≤ 5e-8). The models were adjusted for age, sex, education, baseline MMSE (not for the intercept), *APOE* ε2 and ε4 count, and the top 10 principal components from the principal component analysis on the entire set of genotype data. The y-axis shows the negative log of the *p* value showing the significance of association for PRS models with different β-amyloid measures. The values on the top of each bar show the association's effect size (β-coefficient). For negative effect size, the bar is inverted. CSF Aβ42/Aβ40 is used as a dichotomous variable here (with 1 = Aβ positive). The horizontal dotted line shows the *p* value threshold of 0.05. Aβ = β-amyloid; MMSE = mini‐mental state examination; PRS = polygenic risk score; P-tau181 = phosphorylated tau181; T-tau = total tau.

Next, we tested associations between PRS and Aβ biomarker measurements (Aβ1-38, Aβ1-40, Aβ1-42, and Aβ42/Aβ40 ratio) in BioFINDER. Owing to its bimodal distribution, the Aβ42/Aβ40 ratio was used as a dichotomous rather than a continuous variable.^20^ Non-*APOE* PRS2 and PRS4 had nominally significant associations with Aβ42/Aβ40, but no associations were significant after Bonferroni correction (Figure 2; eTables 4–7, links.lww.com/WNL/C21).

All the *APOE*-PRSs showed significant association with the Aβ1-42 and Aβ42/Aβ40 ratio (*p* < 6.2e-09 and *p* < 1.2e-05, respectively). However, there was no significant association between *APOE*-PRSs and Aβ1-38 and Aβ1-40 (eTables 4–7, links.lww.com/WNL/C21).

### Associations Between PRS and NfL

There were no significant associations between tested non-*APOE*-PRSs and CSF NfL levels. However, we found all the *APOE*-PRSs showing significant association with CSF NfL (*p* < 2.8e-02) (eTable 8, links.lww.com/WNL/C21).

### Association Between PRS and Tau Measures Adjusted for Aβ Status

**Figure 3:**
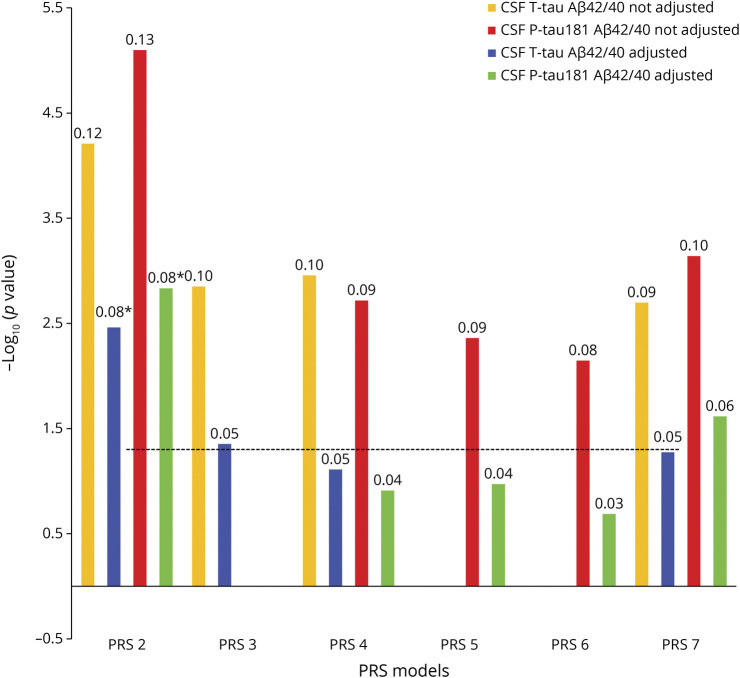
Associations Between Significant PRSs and Tau Measures Adjusted for CSF Aβ42/Aβ40 Ratios The x-axis shows the different PRS models (this analysis only included models that were significantly associated with tau measures when not adjusted for CSF Aβ42/Aβ40 ratios, Figure 1). The models were adjusted for age, sex, education, baseline MMSE (not for the intercept), *APOE* ε2 and ε4 count, and the top 10 principal components from the principal component analysis on the entire set of genotype data, as well as CSF Aβ42/Aβ40 ratios. The y-axis shows the negative log of the *p* value for the significance of associations between PRS models with different tau measures. The values on the top of each bar show the association's effect size (β-coefficient). The horizontal dotted line shows the *p* value threshold of 0.05. *These PRSs were significant after adjusted for CSF Aβ42/Aβ40 ratios and Bonferroni correction at *p* value < 0.05. Aβ = β-amyloid; MMSE = mini‐mental state examination; PRS = polygenic risk score; P-tau181 = phosphorylated tau181; T-tau = total tau.

**Figure 4:**
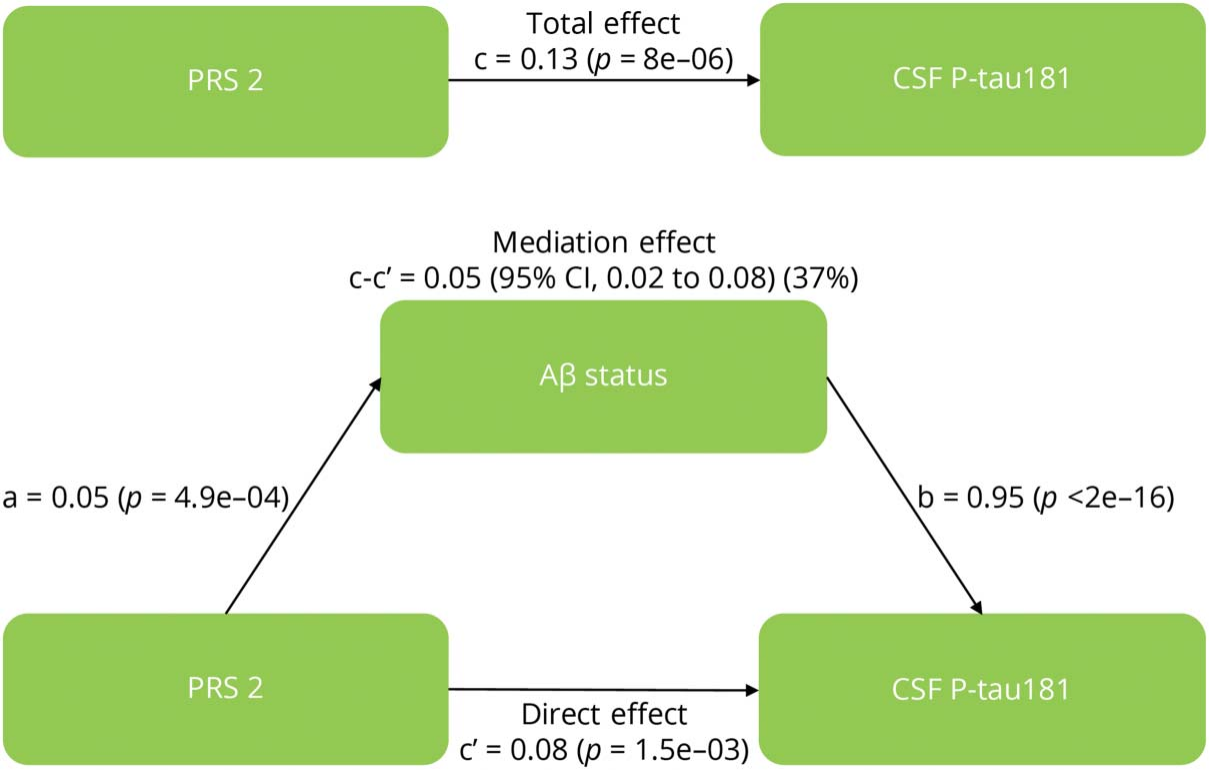
Mediation Analysis Between PRS, Aβ Status, and CSF P-tau181 Mediation analysis with PRS2 as a predictor of CSF P-tau181, mediated by Aβ status. This figure includes the following standardized regression coefficients: a, the effect of PRS on Aβ; b, the effect of Aβ on the CSF P-tau181 level; c, the direct association between PRS and CSF P-tau181 level; c', the association between PRS and CSF P-tau181 level when adjusting for Aβ; and c-c', the mediated effect on the CSF P-tau181 level (with % mediation). Aβ = β-amyloid; PRS = polygenic risk score; P-tau181 = phosphorylated tau181.

To test whether the PRS associations with tau measures were dependent on Aβ, we reperformed the analysis for associations with tau measures for the significant non-*APOE*-PRSs while adjusting for the CSF Aβ42/Aβ40 ratios in BioFINDER. PRS2 was still significantly associated with CSF T-tau (*p* = 1.4e-02) and P-tau181 (*p* = 7.3e-03) (Figure 3; eTables 9 and 10, links.lww.com/WNL/C21), but the strength and significance level of the association were attenuated after adjusting for Aβ status. We, therefore, conducted a mediation analysis to determine the degree to which Aβ mediated the effect of PRS2 (being the most significant non-*APOE*-PRS predicting CSF P-tau181) on CSF P-tau181 levels, a well-studied biomarker for altered metabolism of soluble tau in AD.^28^ As a result, the association between PRS2 and levels of CSF P-tau181 was mediated in part (37%) by Aβ positivity (Figure 4; eTable 11). We also found that the association between PRS4 and CSF P-tau181 was 40% mediated by Aβ positivity (eTable 11).

### Stratified Analysis Based on Clinical Status

We performed subgroup analyses to test the association between PRS and CSF biomarker levels in the CU, MCI, and AD groups. In CU, the non-*APOE*-PRS2 had significant associations with CSF T-tau (*p* = 2e-02; eTable 12, links.lww.com/WNL/C21) and P-tau181 (*p* = 6.5e-03; eTable 13). None of the non-*APOE*-PRSs in any group had a significant association with CSF Aβ biomarkers or NfL (eTables 14–18).

The *APOE*-PRS2 to PRS7 in the CU and MCI groups had significant associations with CSF T-tau (*p* < 1.8e-06 and *p* < 2e-02, respectively; eTable 12, links.lww.com/WNL/C21), P-tau181 (*p* < 4.2e-06 and *p* < 4.1e-02, respectively; eTable13), Aβ1-42 (*p* < 8.4e-05 and *p* < 1.2e-02, respectively; eTable 14), and Aβ42/Aβ40 ratio (*p* < 2e-06 and *p* < 7.6e-04, respectively; eTable15). *APOE*-PRS1 was associated with CSF Aβ1-42 in CU (*p* = 3e-02). We did not find any significant association between the *APOE*-PRSs and CSF Aβ1-38, Aβ1-40 and NfL (eTables 16–18).

### Stratified Analysis Based on *APOE*-ε4 Status

We performed another stratified analysis based on *APOE*-ε4 status (negative = 0 ε4 alleles; positive = 1–2 ε4 alleles). In the *APOE*-ε4 positive group, PRS2-7 had significant associations with CSF T-tau (*p* < 1.2e-03 [non-*APOE*-PRS] and *p* < 1.4e-03 [*APOE*-PRS]; eTable 19, links.lww.com/WNL/C21) and P-tau181 (*p* < 1e-02 [non-*APOE*-PRS] and *p* < 3.6e-05 [*APOE*-PRS]; eTable 20). *APOE*-PRS1 was significantly associated with CSF P-tau181 (*p* = 4.7e-03) in the *APOE*-ε4 positive group. In the *APOE*-ε4 negative group, only PRS2 had significant associations with CSF T-tau (*p* = 3.1e-03 [non-*APOE*-PRS] and *p* = 4.2e-03 [*APOE*-PRS]; eTable 19) and P-tau181 (*p* = 4e-04 [non-*APOE*-PRS] and *p* < 1.4e-03 [*APOE*-PRS]; eTable 20).

In the *APOE*-ε4 positive group, *APOE*-PRS2 to PRS7 were significantly associated with CSF Aβ1-42 (*p* < 1.5e-03; eTable 21, links.lww.com/WNL/C21) and Aβ42/Aβ40 ratio (*p* < 2.9e-03; eTable 22). PRS1 showed significant association with CSF Aβ1-42 (*p* = 1.2e-02 [non-*APOE*-PRS] and *p* = 9.9e-05 [*APOE*-PRS]; eTable 21). Non-*APOE*-PRS4 to PRS6 were found to be significantly associated with the Aβ42/Aβ40 ratio (*p* < 3.8e-02; eTable 22).

In the *APOE*-ε4 negative group, non-*APOE*-PRS2 was found to be significantly associated with the Aβ42/Aβ40 ratio (*p* = 3.1e-02; eTable 22, links.lww.com/WNL/C21). However, there was no significant association between any PRSs and CSF Aβ1-38, Aβ1-40 and NfL in this stratified analysis (eTables 23–25).

### PRS Variants Specific to Tau and Independent of Aβ

The above findings indicated that Aβ pathology partially regulated the effect of PRS2 on CSF P-tau181. We hypothesized that this non-*APOE*-PRS might be heterogeneous, with certain genetic components exerting their influence by the aggregation of Aβ pathology and others acting independently of Aβ pathology on tau metabolism. We investigated non-*APOE*-PRS2 (consisting of 1,742 variants) using a heuristic technique (see Methods section). We found 853 variants whose absence from the PRS strengthened the association between PRS and Aβ compared with the full PRS2 (step 1). We also discovered 890 variants that, when removed from the PRSs, weakened the association between PRS and Aβ compared with the full PRS2 (eTable 26, links.lww.com/WNL/C21). On arranging these PRSs in the ascending order of *p*-value of association between PRS and Aβ, we recreated different PRSs, each with an ascending number of variants (step 2). We identified 79 other PRS models (with an increasing number of components) for which there was no significant mediation by Aβ (on CSF P-tau181). However, these PRSs still predicted CSF P-tau181 significantly (both when adjusting and when not adjusting for Aβ). Among these 79 PRS models, a model containing 1,683 variants (PRS2-Incl-1683) was identified as an optimal Aβ-independent subset because it did not show any difference in the effect size on CSF P-tau181 when adjusted for Aβ (β = 0.08, *p* = 2.3e-03) and when not adjusted (β = 0.08, *p* = 7.5e-03) (eTable 27).

Finally, to identify the subset of the PRS that was likely acting on CSF P-tau181 through Aβ, we constructed a PRS that contained variants that did not overlap with the Aβ-independent PRSs. This PRS model (PRS2-R-Incl-19) included the 19 variants that were not part of any Aβ-independent-PRS (eTable 28, links.lww.com/WNL/C21). We call this PRS model the “Exclusive Aβ-dependent PRS model.” This model had a very similar effect on Aβ (the mediator) (β = 0.14, *p* = 6.6e-21) and on CSF P-tau181 (when not adjusted for Aβ) (β = 0.15, *p* = 7.3e-07). When corrected for Aβ, the model's effect on CSF P-tau181 was markedly reduced and nonsignificant (β = 0.02, *p* = 5.7e-01), supporting that the components included affected CSF P-tau181 through the accumulation of Aβ.

Furthermore, we tested a model with both “PRS2-Incl-1683” and “PRS2-R Incl-19” as predictors of CSF P-tau181, along with the previously used covariates. Both these PRSs were found to be significantly and independently associated with CSF P-tau181 when not adjusting for Aβ status (PRS2-Incl-1683: β = 0.08, *p* = 4.9e-03; PRS2-R-Incl-19: β = 0.15, *p* = 5e-07). After adjusting the analysis for Aβ status, PRS2-R-Incl-19 (as expected) lost the association with CSF P-tau181 (β = 0.02, *p* = 5.2e-01), whereas the association between PRS2-Incl-1683 and CSF P-tau181 was unchanged (β = 0.08, *p* = 2.2e-03) (eTable 29, links.lww.com/WNL/C21).

### Validation in ADNI

**Figure 5:**
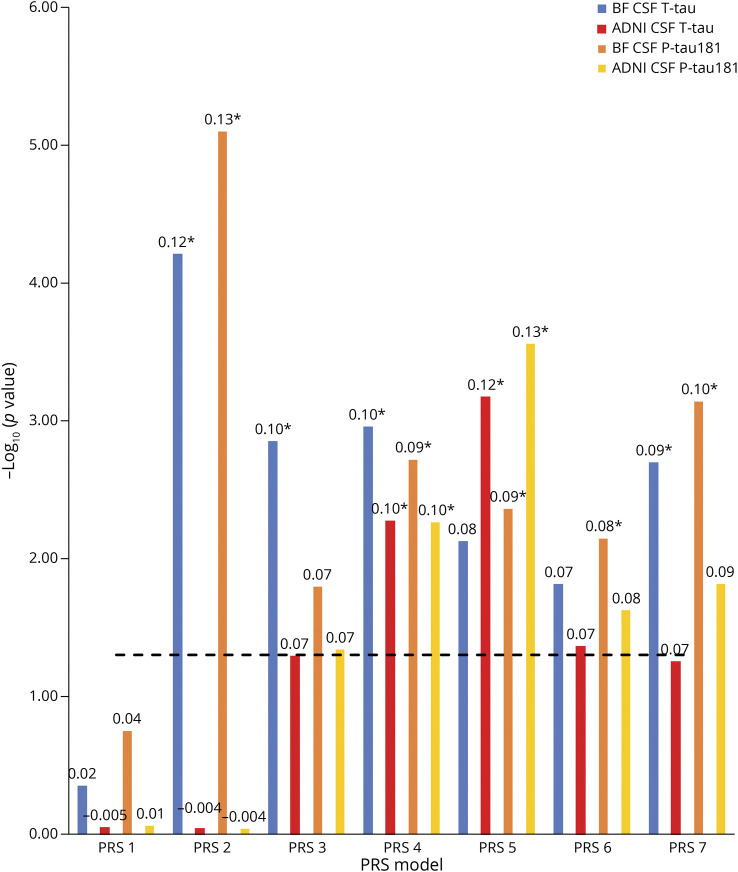
Comparative Results for Associations Between PRSs and Tau Measures in BF and ADNI The x-axis represents the 7 different PRS models at different *p* value thresholds based on the GWAS summary statistics (PRS1 ≤ 0.05, PRS2 ≤ 5e-3, PRS3 ≤ 5e-4, PRS4 ≤ 5e-5, PRS5 ≤ 5e-6, PRS6 ≤ 5e-7, and PRS7 ≤ 5e-8). The models were adjusted for age, sex, education, baseline MMSE, *APOE* ε2 and ε4 count, and the top 10 principal components from the principal component analysis on the entire set of genotype data. The y-axis shows the negative log of the *p* value for the significance of associations between PRS models with different tau measures. The values on the top of each bar show the association's effect size (β-coefficient). The horizontal dotted line shows the *p* value threshold of 0.05. *These PRSs were significant after Bonferroni correction at *p* value <0.05. Aβ = β-amyloid; ADNI = Alzheimer's Disease Neuroimaging Initiative; BF = BioFINDER; MMSE = mini‐mental state examination; PRS = polygenic risk score; P-tau181 = phosphorylated tau181; T-tau = total tau.

We replicated our findings in an independent data set from ADNI using 777 CU, MCI, and AD samples of European ancestry. PRS5 (including 29 SNPs significant at *p* < 5e-06) showed the strongest association with both CSF T-tau (*p* = 4.7e-03) and CSF P-tau181 (*p* = 1.9e-03) after applying the Bonferroni correction for multiple comparisons. It was followed by PRS4 (including 80 SNPs significant at *p* < 5e-05), showing significant associations with CSF T-tau (*p* = 3.7e-02) and CSF P-tau181 (*p* = 3.8e-02) (Figure 5; eTables 30 and 31, links.lww.com/WNL/C21). PRS7 (including 10 SNPs significant at *p* < 5e-08) was found to be significantly associated with CSF Aβ1-42 (*p* = 3.6e-02) (eFigure 1, links.lww.com/WNL/C23; eTable 32). PRS2 (including 2,185 SNPs significant at *p* < 5e-03), which was associated with the BioFINDER tau measures, did not show significant associations with ADNI tau measures (CSF T-tau [*p* = 9e-01] and CSF P-tau181 [*p* = 9.2e-01]) (eTables 30 and 31).

We also tested for PRS associations with tau measures while adjusting for CSF Aβ1-42 to see whether the association for significant PRSs (PRS4 and PRS5) was independent of Aβ. We observed a nominal increase in the association *p*-value for PRS4 and PRS5 with CSF T-tau (*p* = 4.5e-02 and 6.7e-03, respectively) and P-tau181 (*p* = 4.6e-02 and 2.8e-03, respectively). Still, the effect size remained unchanged in both the Aβ adjusted and unadjusted analyses (eFigure 2, links.lww.com/WNL/C23; eTables 33 and 34, links.lww.com/WNL/C21), indicating that PRS4 and PRS5 for ADNI participants were tau-specific and independent of Aβ.

In addition, we also conducted a mediation analysis to determine the extent of Aβ mediation on PRS4 predicting CSF P-tau181 levels in ADNI. Our result indicated that the association between PRS4 and levels of CSF P-tau181 in ADNI was not mediated by Aβ positivity (eFigure 3, links.lww.com/WNL/C23; eTable 35, links.lww.com/WNL/C21). This analysis further confirmed that the PRS4 for ADNI participants was tau-specific and independent of Aβ.

## Discussion

We investigated whether a priori defined PRSs for AD (characterized by contrasting AD dementia vs controls) were associated with different levels of AD-related fluid biomarkers in a cohort with participants ranging from CU to MCI and AD patients. Our main findings were that PRSs (beyond the *APOE* region variant) for AD were associated with higher levels of CSF tau biomarkers (with most substantial effects for comparatively inclusive PRSs) rather than biomarkers of Aβ and neurodegeneration. The same was true within CU and MCI groups when stratified by clinical status. Taken together, these findings suggest that AD-associated PRS models are related to pathophysiologic changes of AD, including altered tau metabolism, such as increased neuronal production and secretion of tau.

The PRS analyses revealed a significant relationship between an overall greater load of AD-associated genetic risk factors beyond *APOE*, measured by the PRS-metric, and increased CSF T-tau and P-tau181. These results indicate that the genetic profile contained within the PRSs modulates AD pathogenesis in tau metabolism. A recent finding^29,30^ showed that soluble P-tau (plasma or CSF) is very closely related to Aβ pathology and mediates the effects of Aβ on tau tangles. Therefore, increased production, phosphorylation, or secretion of tau (caused by Aβ) might be essential for the development of tau tangles and, later on, neurodegeneration. Our results show a strong association of AD-related PRS with increased P-tau. This genetic evidence suggests that the increased extracellular levels of tau may be an important drug target in AD.

We also observed that the association between PRS and tau markers (CSF T-tau and P-tau181) remained when adjusting for Aβ42/Aβ40. This suggests that the genetic risk factors in the PRS affect tau metabolism through mechanisms that are partly independent of Aβ pathology. We even isolated a subset of PRSs, which seemed to be utterly independent of Aβ (PRS2-Incl-1683). These findings may suggest differential underlying biological mechanisms that could be targeted to affect and prevent pathologic metabolism of Aβ and tau, respectively. Furthermore, since pathologic changes in AD start 15–20 years before clinical presentation^31^ and clinical trials are increasingly focused on early, even preclinical, disease stages,^32^ such mechanisms may be relevant to also target very early in individuals who only have mild or no cognitive impairment.

Our results on PRS and AD biomarkers extend the knowledge in a field where previous studies have presented somewhat mixed results. Some studies have, like us, found associations between PRSs (or polygenic hazard scores, PHSs) and AD biomarkers. In recent analyses in the ADNI cohort, PHSs for AD were associated with CSF T-tau and P-tau181^33^ and plasma P-tau181,^34^ and these associations were independent of *APOE*. One of these studies^33^ also reported a nominal association level between PHS and CSF Aβ. Another study with CU and MCI individuals found that PHS was associated with CSF Aβ and CSF T-tau.^35^ These results are comparable with and support our results for associations between PRS and CSF biomarkers. A PRS study on patients with MCI from 4 European cohorts also reported a similar finding to ours using CSF Aβ, T-tau, and P-tau181.^36^ Associations between PRS and CSF T-tau and P-tau181 were reported in a study with only patients with AD, but the same study could not establish an association with Aβ (for a PRS without *APOE*).^37^ A study using the European Medical Information Framework Alzheimer's Disease Multimodal Biomarker Discovery data reported a significant PRS association with CSF Aβ1-42 but not with CSF T-tau and P-tau levels.^38^ A study based on a predementia (MCI) sample of participants showed a significant association with CSF Aβ1-42 and minimal associations with CSF T-tau and P-tau, which is in line with the existing evidence that T-tau and P-tau are later markers of AD compared with Aβ measures.^39^ The Australian Imaging, Biomarkers and Lifestyle study of aging showed no correlation between PRS and either of the CSF T-tau, P-tau, or Aβ levels in a smaller (570 patients with CU and 73 patients with AD dementia) sample,^40^ possibly because of fewer individuals with AD pathology in the sample. Some of the differences between studies may reflect different statistical power to detect effects.

In some cases, this could be driven by overly homogenous populations with a restricted range of biomarker levels. Differences in the disease stage, and sometimes even differences in SNPs included in the PRSs, are other potential explanations for the different results in different cohorts. However, in summary, the well-powered studies that take in the full range of AD from preclinical to MCI and dementia stages seem to demonstrate that AD-related PRSs are associated with biomarker changes reflecting both abnormal tau and Aβ metabolism. Some biomarkers, however, do not seem to be strongly regulated by SNPs (beyond the *APOE* region), which is evident from the comparative result of association of the PRS models generated by including and excluding *APOE* region variants. However, the same biomarkers showed a stronger association with the PRS models (in full as well as stratified analysis by clinical status) generated using *APOE* region variants.

The exact relationship between Aβ and tau pathologies in AD is still unclear. Our analyses identified partly Aβ-independent genetic pathways to tau pathology. However, other studies have suggested that tau mediates Aβ toxicity, for example, by interacting with Fyn kinase through its amino-terminal projection domain.^41^ This may open for the hypothetical possibility that pathologic tau could mediate a relationship between risk genes and Aβ pathology. However, since we did not find any significant associations between non-*APOE*-PRSs and CSF Aβ (when correcting for multiple comparisons), we could not test whether tau mediated effects of genes on Aβ. Larger studies or studies focusing on specific relevant genes may be needed for this.

We found the most robust results for non-*APOE*-PRS2 in BioFINDER and conducted detailed analyses of gene enrichment in PRS2 and the 2 restricted PRSs (Aβ-independent-PRS and Aβ-dependent-PRS) (detailed results and discussion in the eMethods, links.lww.com/WNL/C22; eTables 36–44, links.lww.com/WNL/C21; eFigures 4-7, links.lww.com/WNL/C23). The gene ontology (biological process) term “amyloid-beta clearance” was enriched in the overall PRS2, but not in the restricted Aβ-independent-PRS, further confirming that this restricted PRS might be tau-specific and Aβ-independent.

For the Aβ-dependent-PRS set, 2 terms (“amyloid plaque” and “amyloidosis”) were explicitly enriched. Enrichment of these 2 terms specifically for this PRS supports our finding that the genes involved contribute toward abnormal Aβ formation. These results confirm and strengthen the use of this PRS to study Aβ-dependent genetic effects (beyond the *APOE*) on tau metabolism.

Although we could not establish any association between non-*APOE*-PRS and NfL, a recent study found an association between non-*APOE*-PRS and NfL in individuals without Aβ1-42 pathology.^42^ Our analysis was not stratified by measures of Aβ pathology. Future studies may continue to elucidate associations between PRS and NfL.

Using the independent ADNI cohort, we could replicate the associations for PRS4 with CSF T-tau and P-tau181, and for PRS5 with CSF P-tau181. It is important that these associations in ADNI were independent of Aβ status, supporting the findings in BioFINDER that the genetic pathways regulating CSF tau metabolism are largely independent of Aβ. However, we could not validate the PRS2 association with tau measures. There are several possible reasons why PRS2 was not validated in ADNI. First, variants with identical effect sizes may have different allele frequencies across populations, which would result in heterogeneous allele substitution effects. Second, PRS2 has a large genetic diversity (constructed using 1,742 variants in BioFINDER and 2,185 variants in ADNI), introducing variability. Third, using different gene-centering genotyping platforms for the data sets might cause this discrepancy. Another possibility is that the varying and relatively small sample size of unique individuals in both the cohorts could have influenced the total number of variants with the good quality available after the imputation.

Our study is not without limitations. Although the BioFINDER cohort has robust phenotyping for CSF tau and Aβ biomarkers, the sample size was comparatively small. This could be one of the reasons that we were unable to detect associations between non-APOE-PRS2 and CSF T-tau and P-tau181 in the AD and MCI groups (smaller sample sizes than the CU group). Owing to the small sample size, we just included gene variants with MAF >0.05. A larger sample size may account for rarer SNPs and make findings stratified for APOE or clinical status more interpretable. The study also has strengths. The BioFINDER cohort reflects a population of consecutively recruited patients and healthy controls who are less selected than trial-like populations (e.g., ADNI), which supports the generalizability of the findings. In addition, the use of a priori defined PRSs partly overcome the issue of multiple testing by integrating many SNPs into a small number of metrics of different complexity.

In conclusion, our results extend the knowledge about the relationship between genetic risk for AD beyond *APOE* and AD-related biomarkers. Our stratified analysis based on the *APOE* genotype showed stronger associations in the *APOE*-ε4 positive group for all the PRS with the CSF biomarkers (CSF T-tau, P-tau181, Aβ1-42, and Aβ42/Aβ40 ratio). This suggests that our genetic findings are independent of APOE-ε4. Our findings suggest that integrating PRS models with biomarker data holds promise for understanding genetic pathways linked to disease development. Future directions also include testing interactions between SNPs, biomarker levels, and disease stage to understand how SNPs may affect disease processes at different time points during the disease development. Genetic studies may also be performed using longitudinal biomarker data. Finally, although our results mainly point to genetic effects at the group level, future studies may test whether specific SNPs can be combined with biomarker data to improve the subject-level management of patients in clinical practice and clinical trial design.

## Glossary

Aβ: β-amyloid
AD: Alzheimer disease
ADNI: Alzheimer's Disease Neuroimaging Initiative
CU: cognitively unimpaired
GWAS: genome-wide association study
MAF: minor allele frequency
MCI: mild cognitive impairment
NfL: neurofilament light
PHS: polygenic hazard score
PRS: polygenic risk score
P-tau: phosphorylated tau
QC: quality control
SNP: single-nucleotide polymorphism
T-tau: total tau
